# The great urban shift: Climate change is predicted to drive mass species turnover in cities

**DOI:** 10.1371/journal.pone.0299217

**Published:** 2024-03-27

**Authors:** Alessandro Filazzola, Marc T. J. Johnson, Kimberly Barrett, Sue Hayes, Namrata Shrestha, Laura Timms, James Scott MacIvor

**Affiliations:** 1 Centre for Urban Environments, University of Toronto Mississauga, Mississauga, Ontario, Canada; 2 Apex Resource Management Solutions, Ottawa, Ontario, Canada; 3 Department of Biology, University of Toronto Mississauga, Mississauga, Ontario, Canada; 4 Conservation Halton, Burlington, Ontario, Canada; 5 Toronto and Region Conservation Authority, Concord, ON, Canada; 6 Department of Watershed Knowledge, Credit Valley Conservation, Mississauga, Ontario, Canada; 7 Department of Biological Sciences, University of Toronto Scarborough, Toronto, Ontario Canada; University of Agriculture Faisalabad, PAKISTAN

## Abstract

Human experiences with nature are important for our culture, economy, and health. Anthropogenically-driven climate change is causing widespread shifts in biodiversity and resident urban wildlife are no exception. We modelled over 2,000 animal species to predict how climate change will impact terrestrial wildlife within 60 Canadian and American cities. We found evidence of an impending *great urban shift* where thousands of species will disappear across the selected cities, being replaced by new species, or not replaced at all. Effects were largely species-specific, with the most negatively impacted taxa being amphibians, canines, and loons. These predicted shifts were consistent across scenarios of greenhouse gas emissions, but our results show that the severity of change will be defined by our action or inaction to mitigate climate change. An impending massive shift in urban wildlife will impact the cultural experiences of human residents, the delivery of ecosystem services, and our relationship with nature.

## Introduction

Nature is an integral element of cities globally. Over half the world’s population live in cities and the wildlife that people observe within their respective urban realm represents the species with which they have the most direct familiarity [1, 2]. We value these urban species because they provide a benefit in terms of delivering ecosystem services, such as supporting mental well-being, providing pollination or pest removal, and recreation [3–6]. Iconic species can also be emblematic of the community within cities [7], such as the animal species used as mascots for sports teams or represented on governmental flags. However, anthropogenic impacts such as climate change can threaten the presence of species in cities [8], making iconic and familiar species at risk of extirpation from the communities they represent. Just like the California grizzly bear is extinct from where it is displayed prominently on the state flag, with climate change, the floodgates are open and many other emblematic species are at risk of extirpation from the communities they represent [9, 10]. In other instances, gradual changes in species composition can go unnoticed between generations of human residents because of changing expectations of what constitutes the natural environment, i.e., the shifting-baseline syndrome [11–13]. Thus, future generations of urban dwellers may be unaware that the wildlife they experience in their home cities is different than what exists today. Alternatively, the shift of urban species may be so substantial and within a single generation that it will be clearly noticeable among residents.

Anthropogenically-driven climate change is threatening species globally [14, 15], and cities are no exception. There has been repeated evidence that climate change will cause widespread shifts in a range of species and from all types of taxa [16–19]. While climate change is moving species across the continents (e.g., poleward and into higher elevations) [18–21], city boundaries are relatively fixed in space and are therefore likely to undergo climate driven changes in biodiversity patterns. For instance, common migratory songbirds in backyards have begun moving poleward in response to warming winter temperatures in North American cities [22]. Certain bioregions will also have greater vulnerability to climate change, including areas of North America where many major cities are located—such as temperate mixed forests and boreal coniferous forests [23]. Within the coming decades, we may observe significant species turnover (i.e., changes in the abundances and occurrence of species) in some areas as rapid climate change affects community assembly and species dispersal [10, 24]. As a result, an individual who lives a lifetime within the same city will likely observe changes in the species that occur around them. Some research has already projected significant changes in the composition of urban plants and bird species for European cities in the next 60 years [25, 26]. However, an examination of the potential shifts in community composition from climate change for all animal taxa in cities has not been comprehensively conducted in North America.

Here, we provide a synthesis of the extent that climate change is anticipated to have on biodiversity within cities. We hypothesized that climate change will drive a significant turnover in the composition of urban species in Canadian and American cities causing a *great urban shift* by the end of the century as species ranges track shifting temperature and precipitation patterns. We modelled the historic and future species distributions for 2,019 terrestrial animal species found in 60 cities in Canada and the United States. These 60 cities represent highly developed urban areas each with a population over 400,000 in the core municipal area (S1 Table). We selected species based on the frequency of verified observations per city (i.e., n > 10 individuals per city) by researchers and community scientists. Future climate models included an ensemble of six global circulation models (GCMs) and under three shared socio-economic pathways (SSPs) predicted until the end of the century (2081–2100). We compared the change in predicted occurrence of species based on climate suitability between historical and future climates to determine the species and cities that are expected to be most affected. Although it was not the original motivation for our study, our analyses allowed us to compare the differences in species native status (i.e., native vs. exotic) and IUCN Red List status (https://www.iucnredlist.org/), since these species have important conservation implications.

## Methods

### City and species selection

We chose the 60 most populated cities in Canada and the United States, which all have populations over 400,000 people (S1 Table). In each of these 60 cities, we created a 20 x 20 km quadrat around the centroid of the municipal boundary. For consistency, we picked this quadrat size for all cities regardless of the municipal boundaries to capture the core urban areas of selected cities. The size of this quadrat also minimized placement outside of the city boundaries or in large waterbodies. Using the Global Biodiversity Information Facility (GBIF; https://www.gbif.org/), we downloaded all species records for terrestrial animals found within that quadrat. All records of species occurrences used and their associated databases can be found at S2 Table. The term “terrestrial” here is meant to represent animals that do not spend their entire life cycle in water (e.g., fish, cetaceans) and thus would include semi-aquatic organisms (e.g., amphibians, dragonflies) and flying organisms (e.g., bats, birds). Species records were filtered to include all animal species that have at least ten records within the last ten years for any of the 60 cities, indicating the species has been observed enough times that it was not incidental. Many target taxa were observed in multiple cities, such as hawks (*Accipiter* spp., Accipitridae), dabbling ducks (*Anas* spp., Anatidae), and bumble bees (*Bombus* spp., Apidae*)* but some species were found unique to only one city, such as the bark anole lizard (*Anolis distichus*) in Miami or Strand’s carpenter bee (*Xylocopa strandi*) in Houston. There was a bias in the species list towards taxa that are larger and more identifiable, as is typically found in community science, but also in traditional science [27].

In total, we found 2,259 unique species that matched our criteria. For each of these species, we used GBIF to download all occurrences between 2000 and 2020 for all North America. We selected this area, larger than Canada and the USA where our selected cities are present, to capture the total climatic niche and range of conditions that each selected species can occupy. In total, we downloaded over 18.4 million occurrence records from GBIF with a median of 1,059 records per species (minimum 10 records, maximum 138,746 records). Although there were large differences in records per species, our modelling approach was robust to infrequently surveyed species [28, 29] such that similar confidence could be treated among model results.

There have been reported issues with the reliability of GBIF data concerning the accuracy of records in time, space, and species identification [30, 31]. While no one approach can be applied to solve all issues associated with GBIF records [30], steps can be taken to minimize the impact and increase confidence [32]. We recognize that the size of our dataset makes verification of every individual record impractical, and thus despite our efforts, some amount of inaccuracy will remain. For all records, we restricted occurrence to North America, which removes common errors associated with coordinates labelled as zero or mistakenly entered records (e.g., latitude and longitude swapped). Our analysis was not reliant on time, therefore temporal issues, such as mismatches in months or days, would not be impactful on our results. We removed all records in the oceans and removed duplicates. Removing duplicates will also mitigate issues such as when records are reported as the centroid or capital of a country since, if inaccurate, would only represent one out of potentially thousands of records. Similarly, inaccuracies in species identification may remain within the dataset, but we expect that the occurrence of relatively few incorrect methods would have a small impact on our large dataset distributed across Canada and the US.

### Climate variables

We used a series of future climate models to capture the range of potential outcomes for the end of the century (2081–2100) under different greenhouse gas emission scenarios. All data climate models, data management, and statistical analyses were conducted in R Version 4.1.0 [33]. We downloaded 24 bioclimatically relevant variables from ClimateNA [34, 35] that represent down-scaled climate variables in 4.6 km grid cells. In addition to the current climate conditions (1990–2020), we also downloaded an eight-model ensemble of future climate condition [34]. These models were all selected under the Coupled Model Intercomparison Project Phase 6 (CMIP6) and include the global circulation models (GCM) that are more representative of the North American climate [34]. Using an ensemble model provides a more conservative estimate of climate change effects on species distributions because it reduces model-specific anomalies [36]. We downloaded the future climate conditions for 2081–2100 under three shared socioeconomic pathways (SSP 1–26, SSP 3–70, SSP 5–85). We selected the three SSP scenarios to represent a range of outcomes based on action to reduce greenhouse gas emissions including sustainable development (SSP 1–26), barriers to mitigating climate emissions and a lack of regional cooperation (SSP 3–70), and continued development of fossil fuels and land (SSP 5–85) [37]. These SSPs represent the latest framework for future climate projections that considers uncertainty in both the climate outcomes from greenhouse gas emissions (i.e., Representative Concentration Pathways; RCPs) [38] and socioeconomic development in the absence of policies to mitigate climate change [37]. In North America, SSP 1–26 and SSP 5–85 both project increased urbanization although for different reasons with the former under high density development and the latter under increased urban sprawl [39]. The SSP 3–70 projects a relatively little land cover change to urban [39].

### Species distribution modelling

We conducted species distribution modelling for each species to determine the historic climatic niche and use these models to predict their future range. For each species, we conducted corrections for survey bias, minimized spatial autocorrelation, and automated model tuning to quantify the relationship with climate. We used Maximum Entropy (MaxEnt) [40] because our data represents presence-only data and thus requires the generation of pseudo-absences [41]. MaxEnt is a machine learning algorithm that predicts the suitable conditions for a species by modelling the relationship of occurrence records to a set of environmental variables [40]. The GBIF occurrence records are collated from a series of community science sources (e.g., iNaturalist, eBird) and museum specimens. These records typically have unequal sampling efforts favouring areas with greater accessibility such as along roads and in parks, as well as under sampling in difficult-to-access areas such as mountains [42, 43] and private property. To account for unequal sampling, we conducted two methods for bias correction: spatial thinning and restricting background points. Spatial thinning is one of the most effective methods for accounting for sampling bias in MaxEnt [44] and involves removing multiple observations within a certain distance to approximate a systematic sampling of the target species. We spatially thinned our dataset by overlaying a 25 x 25 km raster (i.e., 5 factor larger) and by removing multiple occurrences within the same cell. We also restricted the background records (i.e., pseudo-absences) which has been observed to improve MaxEnt performance when the occurrences occupy an area smaller than the total study area [45].

Using the randomly generated background points, spatially filtered occurrence records, and climate variables without collinearity, we conducted MaxEnt modelling for each species. Since MaxEnt is a presence-only analysis, background points (i.e., pseudo absences) need to be generate in a manner that accurately captures climate conditions with the geographic study area. These background points serve to quantify the available climate conditions to be used as a comparative distribution against the climate conditions specific to the presence records. Spatial autocorrelation, the lack of independence between occurrence records, is a frequent problem when working with spatial environmental datasets [46] including species distribution models [47–49]. Without compensating for spatial autocorrelation, species distribution models tend to overestimate the accuracy of the model and suggest the results that are more reliable than is true [49]. For details on our methods in calculating background points, conducting spatial filtering, and removing collinear variables, see S1 File.

We used an automated tuning and evaluation process for MaxEnt function (*ENMevaluate*, package *ENMeval*) [50]. MaxEnt was automated to assess best model using eight feature classes (L, Q, P, LQ, HQ, QPH, QPHT, and LQHP) and six regularization parameters (0.5, 1.0, 1.5, 2.0, 2.5, 3.0). The acronyms in the feature classes relate to relationship between the predictor variables and the predicted occurrence of the target species including linear (L), quadratic (Q), product (P), hinge (H), and threshold (T) [40, 50]. The regularization parameters control for overfitting by downweighing co-efficients, but must be balanced against preventing model tuning. Tuning was accomplished by using spatial block cross-validation, which splits the target area into a number of grids and then resamples data within each respective grid for training and testing to improve model metrics [50, 51]. Model statistics were then averaged across all spatial subsets. Each species was run with a different combination of feature classes and regularization parameters (48 different models per species) and the best model was selected using the highest average Boyce Continuous index (BCI) value [52, 53]. BCI is ideal for presence-only models because it measures model accuracy based on how the occurrence records differ from a random distribution, with values +1 being accurate, values of 0 suggesting the model is completely random, and values -1 indicating high predictions away from occurrence records. Models were conducted in parallel for efficiency in runtime using GNU parallel [54] on the Compute Canada super computer cluster (www.computecanada.ca/). From the best model determined for each species, we extracted the average training area under the curve (AUC), average BCI, percent contribution of each environmental variable, the optimal feature classes and regularization parameters, and the average difference between training and testing AUC values. We also determined the threshold to cut-off model predictions based on the lowest trade-off between sensitivity and specificity (function *threshold*, package *dismo*). For a visual workflow of the analyses conducted for species distribution modelling, see S1 Fig.

We removed species from further analysis that failed to provide satisfactory model results. For example, a species was not included in the final analyses if there were insufficient records from GBIF to confidently model the distribution (n < 10), if the model failed to produce a best model, or the AUC value was less than 0.70 (240 species removed). All remaining analyses included 2,019 species that met these criteria. For a list of all meta-data associated with modelling for each species including AUC/CBI scores, parameters, and MaxEnt settings, see [55].

### Predicted occurrence based on climate suitability

The output predictions from MaxEnt were fitted to a logistic distribution and represent the predicted occurrence based on climate suitability for the target species to inhabit, and range between 0 (completely unsuitable, low species prevalence) and 1 (ideal climate, high species prevalence). These values can function as a probability that a species may be observed in a city (i.e., 0 = never, 0.5 = occasionally, 1 = often) when considering climate alone. However, we note that this value does not translate to a true probability of occurrence because many non-climate factors could restrict or increase the potential of the species observed (e.g., dispersal, species interactions, resource availability). Additionally, there is some discussion that the logistic output from MaxEnt represents an estimate of the probability of presence, rather than true probability, as the output values are based on user inputs [see 56]. While these considerations of estimating occurrence are especially relevant for determining a species-specific distribution (especially between studies), our study is exclusively examining the relative difference between historic and future estimates of probability within the same species using the same model to predict for both time frames.

We estimated the predicted occurrence of each species for every city under each climate scenario. Within the 20 km quadrat in each city, we created a stratified grid of 100 points that we extracted the historic climate and future climate in each SSP and both timeframes. Using the best MaxEnt model, the predicted occurrence for each of the species was estimated using the extracted climates of the 100 points in each city. If the average predicted occurrence was above the identified threshold from the MaxEnt modelling, we considered that species to occur within the city. Our research question was interested in the relative change in predicted occurrence between future and historical timeframes. Therefore, for all analyses we calculated 1) the number of new, extirpated, and unchanged cities for each species, and 2) the number of gained, lost, and unaffected species for each city (S3 Table).

### Statistical analyses

We tested if there were differences among the three SSP scenarios by conducting two generalized linear models (GLM) with number of gained and lost species per city as the response variables. We fitted each GLM with a negative binomial distribution (package *MASS*, function *glm*.*nb*) because the response variables represented discrete counts that were over dispersed [57]. To test if the number of species historically present related to the future change in composition, we fitted GLMs with predicted gains and losses as the response variables. The SSP scenarios were treated as a predictor. We determined if there were any climatic indicators relating to cities that are either more resilient or vulnerable to projections of climate change by fitting GLMs using mean annual air temperature (MAT) and precipitation (MAP). We used the 1990–2020 average of MAT and MAP for comparisons to changes in species to see which of the current climates was most expected to be affected. Finally, we compared if human population of each city related to predicted changes in contemporary richness by conducted a Pearson correlation test (function *cor*.*test*) using the number of gains and losses associated with each city.

## Results

**Fig 1:**
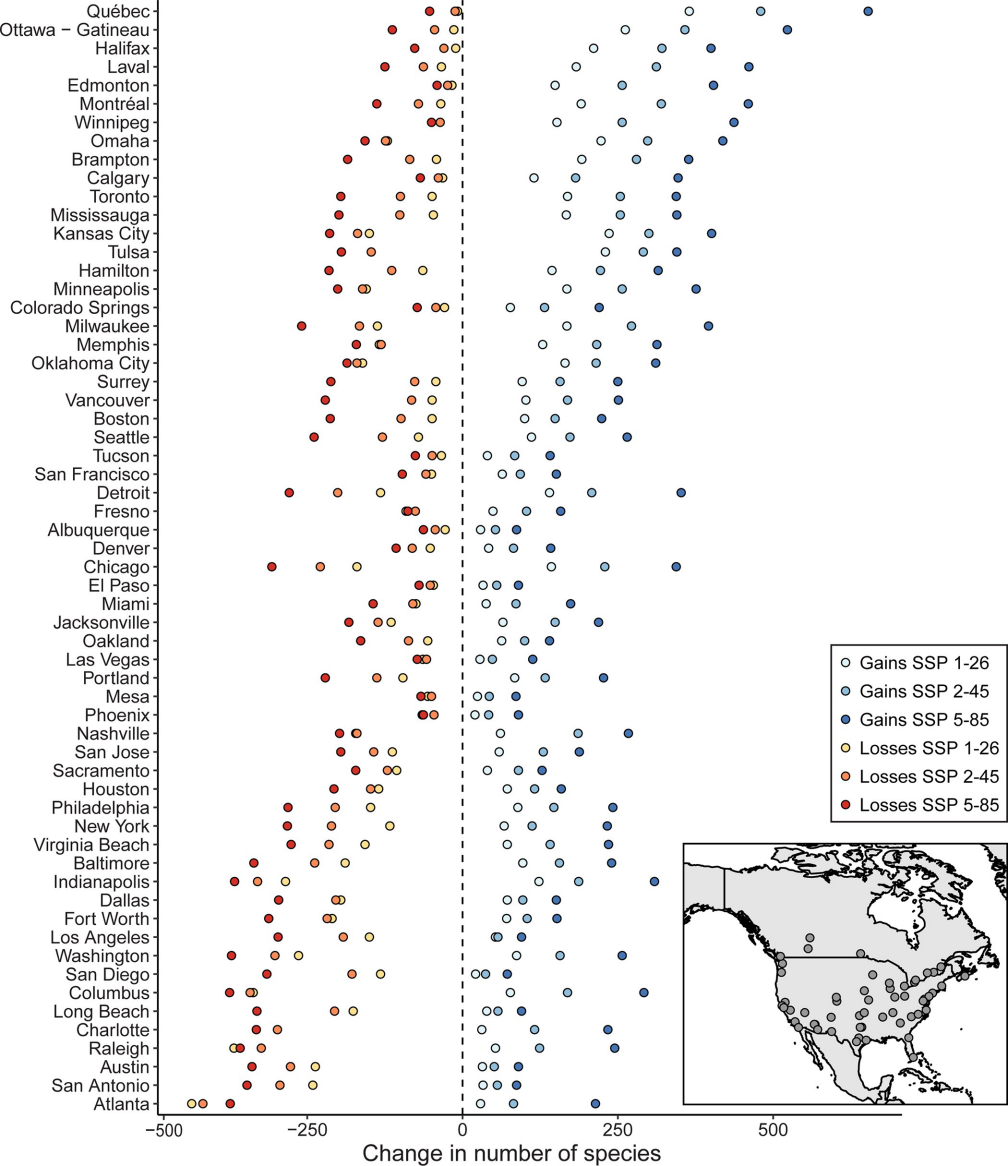
The total number of gains and losses for species in each city separated by SSP scenario. Cities at the top of the figure are predicted to have the greatest increase in species richness relative to species’ historical distribution. Tested cities in Canada and the USA are displayed in the inset map.

The composition of terrestrial animals is expected to significantly shift in many cities by the end of the century (Fig 1). Under all SSP scenarios, every city had both substantial gains and losses of urban species by the end of the century (Fig 1). When exploring cities most sensitive or resilient to changes in composition, we compared mean annual temperatures (MAT) and precipitation (MAP) against the projected changes in species richness. Cities with historically colder temperatures (i.e., MAT < 10° C) were predicted to have significantly higher gains in novel species (MAT: χ^2^_1,178_ = 216.1, p < 0.0001) and fewer losses in resident species (MAT: χ^2^_1,178_ = 21.4 p < 0.0001; S2 Fig). Interestingly, cities with historically high precipitation (MAP > 800 mm) were predicted to have the highest species turnover, with both the greatest gains (MAP: χ^2^_1,178_ = 30.9, p < 0.0001) and largest losses in species (MAP: χ^2^_1,174_ = 45.2, p < 0.0001; S2 Fig). Cities predicted to have the highest introduction of new species (gains > 200 species) included those in temperate Canada, such as Quebec City and Ottawa, and the American Midwest, for example, Omaha and Kansas City (Fig 1). Cities predicted to have the largest species declines (losses > 200) were those in the subtropical eastern parts of the United States and Coastal California (Fig 1). The cities expected to have the fewest changes in contemporary species richness were found in the arid parts of North America, including Las Vegas, Mesa, and Tucson (Fig 1).

We found differences among SSPs where under a scenario of more intense development and greenhouse gas emissions (i.e., SSP 5–85) there were significantly more species lost (χ^2^_2,165_ = 17.6, p = 0.0001; Fig 1) and gained (χ^2^_2,177_ = 62.2, p < 0.0001; Fig 1). For example, depending on SSP scenario, Toronto is predicted to have between 159 and 360 new species occurring within its boundaries by the end of the century while also experiencing a loss of between 40 and 195 species currently present. While this results in a 13.4–18.5% net gain in the number of species, compared to our estimate of 888 species currently predicted for Toronto, these gains and losses represent a massive change in the overall species composition (22% species loss and 41% species gained). We note our estimates only include species with substantial records on GBIF and are not exhaustive accounts of species richness in each city.

**Fig 2:**
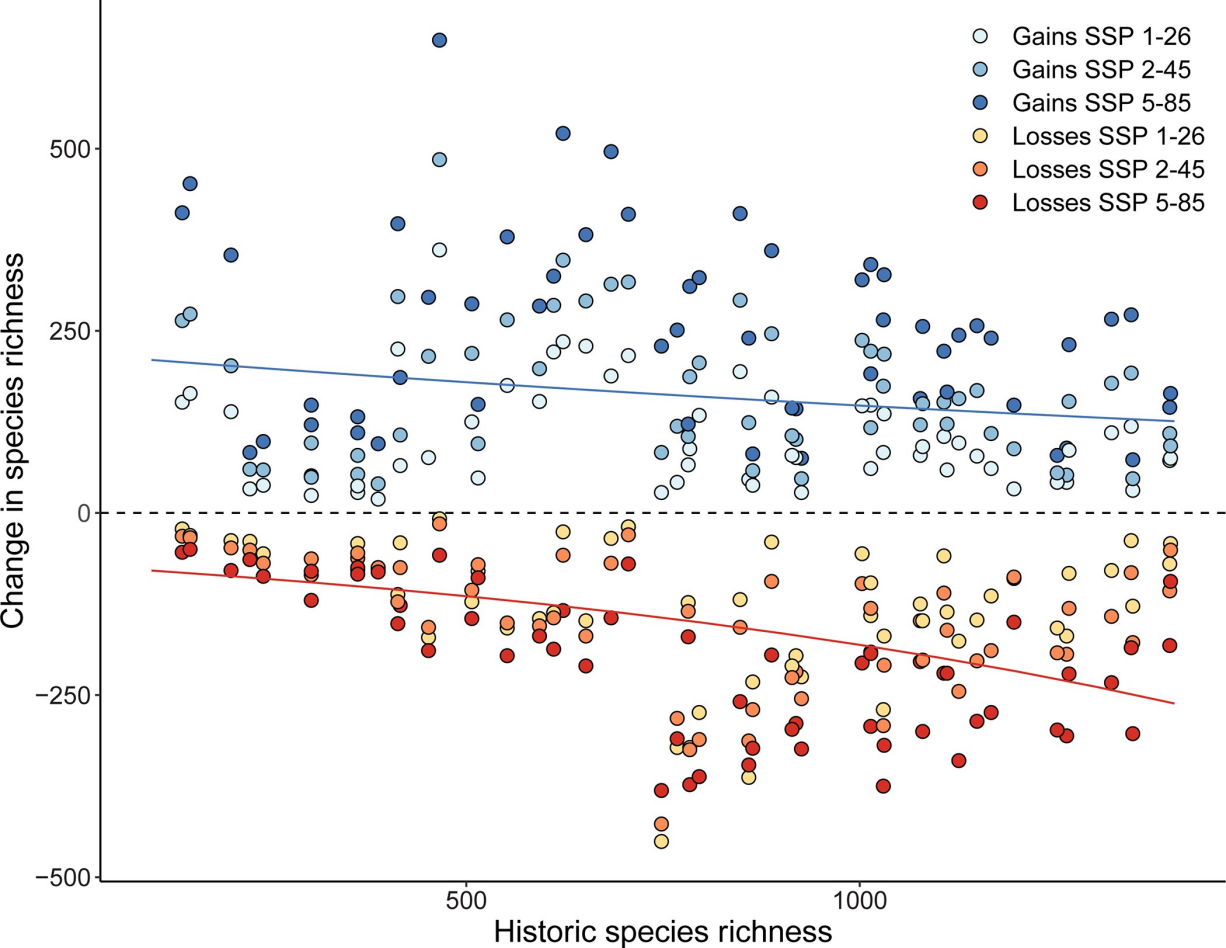
Cities with historically higher species richness were predicted to have significantly more species lost (χ22,165 = 43.0, p < 0.0001) and relatively fewer species gained (χ22,177 = 8.71, p = 0.003) in the future regardless of SSP scenario. Each city is represented six times for each of the three SSP scenarios separated by gains and losses.

Cities with high historic richness were predicted to have the largest declines and fewest gains in species (χ^2^_2_ = 43.0, p < 0.0001; Fig 2). We found that cities with historically lower species richness were anticipated to have significantly higher species gained (χ^2^_2_ = 8.71, p = 0.0031; Fig 2). While these effects were exacerbated under SSP scenarios with greater development and higher greenhouse gas emissions scenarios for both species gained (χ^2^_2_ = 65.3, p < 0.0001) and lost (χ^2^_2_ = 18.4, p = 0.0001), there were no interactions between SSP and historic species richness (loss p = 0.53, gain p = 0.99). We found that city population size was independent of gains (r = -0.06, p = 0.68) and losses (r = 0.18, p = 0.17) in species richness, but some of the most populated cities are predicted to have the greatest declines.

**Fig 3:**
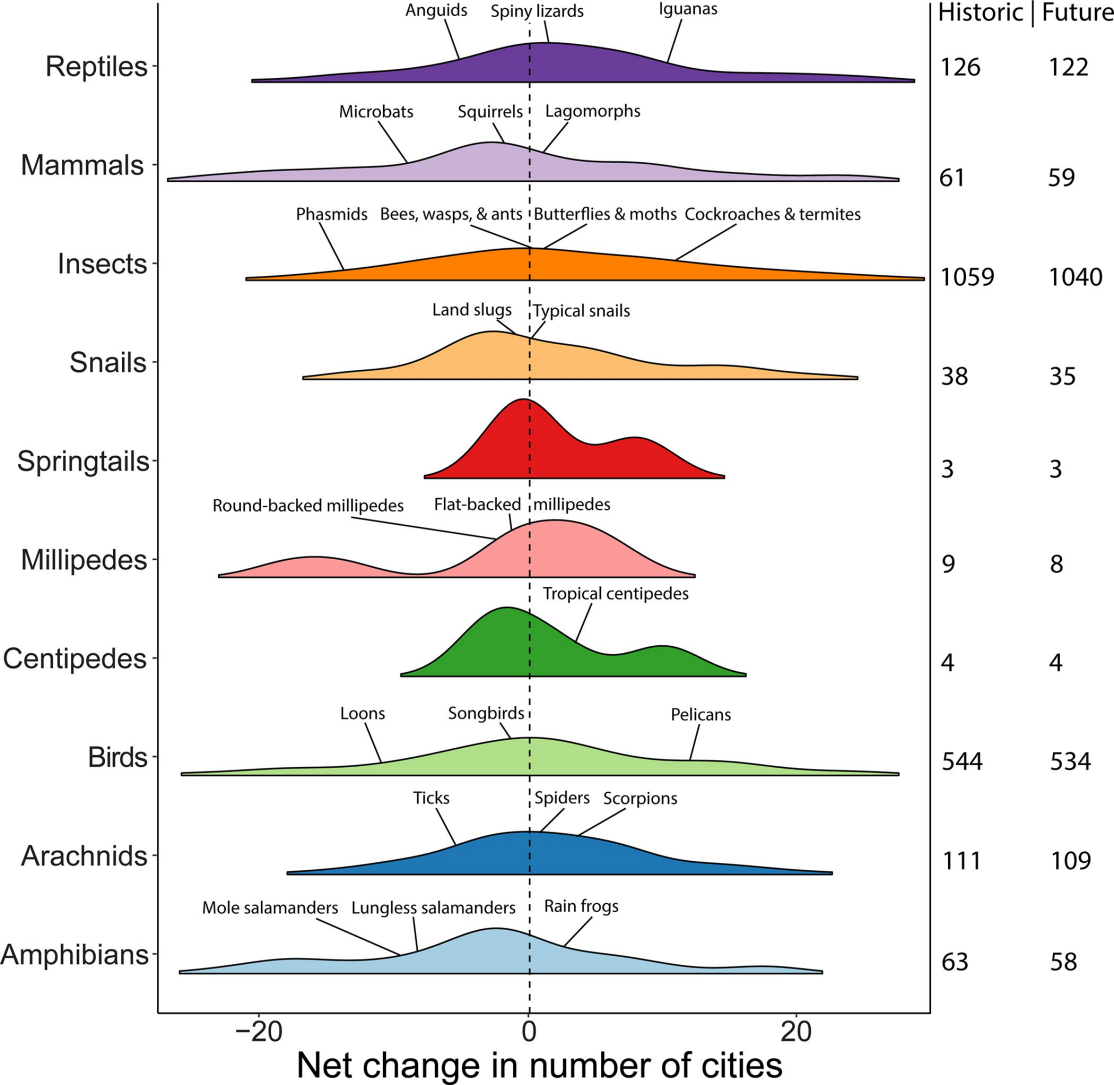
The net change in the number of cities a species will be found in between historic and future climate scenarios for 2,019 animal species separated by class, order, or family. On the right, we present the number of unique species within the respective taxon found within the cities 60 cities in our study for historic and future climate scenarios. Negative values represent a decline in number of cities a species would occupy in the future and positive values represent an increase in the number of cities (i.e., becoming more common). We highlight notable taxa (order or family) with at least two or more species that had extreme values of either large increases or decreases. These notable taxa are presented with their common name and the average net change across all species. The inset map was produced using the GADM administrative boundaries (https://gadm.org/).

Not all species are predicted to be equally impacted by climate change (Fig 3). Among vertebrates, the taxa that on average (among species) were predicted to consistently experience more losses than gains across cities include loons (-28%, Gaviiformes), canids (-17%, Canidae) and anguid lizards (-47%, Anguidae) (Fig 3). Many arthropods were also predicted to decline, including phasmids (-52%, Phasmatodea) and round-backed millipedes (-36%, Spirobolida). Almost all species within the classes of amphibians (-21%, Amphibia) as well as springtails (-11%, Collembola) were projected to decline (Fig 3). Earthworms (-23%, Clitellata) were also predicted to be found in fewer cities, although earthworms were only represented by one species (*Lumbricus terrestris*). Vertebrates predicted to increase in cities included turtles (+59%, Emydidae), mice and other murids (+20%, Muridae), true toads (+38%, Bufonidae) and pelicans (+39%, Pelecaniformes). Some arthropods were also expected to increase, such as net-winged insects (+67%, Neuroptera), scorpions (+26%, Scorpiones), and spiders (+7.5%, Araneae). Although we observed some idiosyncratic responses among species in response to climate change, almost all species (94.5%) experienced some change in the cities where they were found with 44.5% of the species becoming less common in the selected cities and 50% becoming more common. We found that 54 species (2.6%) were predicted to be completely extirpated from all tested cities by the end of the century (Fig 3).

## Discussion

### The great urban shift

Both the predicted species gains and losses are expected to drive widespread turnover of urban biodiversity across nearly all cities in Canada and the United States (Fig 1). Cities with historically cooler temperatures and higher precipitation, such as in temperate Canada and the American Midwest are expected to see the largest influx of novel species. By contrast, relatively hot cities in high precipitation patterns were expected to have the largest loss in resident species richness. These climates are consistent with cities in the subtropical regions of the United States and coastal California, both relatively species rich [58], but expected to have large declines in richness. Our findings coincide with historic species richness correlating to larger species loss and fewer species gains (Fig 2). Cities in the subtropical US, such as Atlanta, have been previously identified as climate sensitive areas and are expected to lose 13.5% of tree species this century [59]. The cities with the smallest predicted changes included those in the arid southwest, such as Mesa, Phoenix, and Albuquerque. While the south western portion of North America is expected to become warmer and drier [58], these ecosystems are believed to be relatively resilient to climate variability when compared to other climates [60], thus limiting the effect of climate change on these cities. Lastly, cities in temperate Canada were expected to see the largest gains in new species and fewest losses (Fig 1) with Quebec, Ottawa, and Winnipeg expecting to nearly double in species richness (Fig 2). The warmer and wetter climate projections for these cities [61, 62] are likely to prove favourable for many animal species currently limited by winter conditions. The response of urban species to climate change is expected idiosyncratic, with certain cities being more sensitive to gains and losses based on contemporary species richness and regional climate patterns.

Greater greenhouse gas emissions and habitat loss will contribute to larger turnover in urban species composition by the end of the century (Fig 1). While our models used climate projects for 2081–2100, the responses of species over the next decades may not be linear. Some species may shift earlier or later depending on tipping points in climate conditions [e.g., 63]. Regardless of actions to mitigate greenhouse gas emissions, substantial shifts are expected to occur in the composition of urban wildlife this century. Climate action to reduce greenhouse gas emissions [64] will determine the extent to which urban species will change in the future. The SSP scenarios were also created with consideration of urbanization rates, with the most rapid and intense urbanization anticipated under SSP1-26 and SSP5-85 [64, 65]. In these scenarios, over 90% of the global population will live in urban areas by the end of the century [64], further emphasizing that in the near future, urban tolerant species will represent the biodiversity people will be most familiar. However, the species affected may be different under the densification of urban development in SSP1-26 compared to the sprawling development of SSP5-85. This raises the debate of land sharing vs. land sparing for urban development to maximize conservation efforts depending on urbanization pattern [11, 66]. Climate change will therefore shape the cultural identity and connection to nature for people in cities.

### Taxonomic responses to climate change

Some of the largest changes in predicted occurrence were observed in birds and insects, which were also the taxa with the largest number of species represented (n = 542 and 1056, respectively). Over 95% of species of birds (49% increase, 46% decrease) and insects (53% increase, 43% decrease) were found to have a change in the number of cities they are predicted to occupy. These results are broadly consistent with a previous study that showed a compositional shift in bird communities visiting urban backyards in North America in recent decades as a result of warming winter temperatures [22]. As a result, future generations of people living in cities may find familiarity with different bird songs than the ones we hear today. Insect biodiversity and abundance is already declining in many regions and urban centres around the world [67–70]. For example, in Raleigh, NC, bee abundance is anticipated to decline 40% per degree of warming [71], a pattern supported by our data predicting a 32% decline in predicted bee species (Anthophila) for Raleigh, as well as a 9% decline in bees across all cities in Canada and the USA. At-risk species as identified by the IUCN Red List were not necessarily more vulnerable to climate change (S2 File), but already have populations in decline from other stressors (e.g., habitat loss, invasive species) that may be exacerbated by climate change. Furthermore, our results show that exotic species had a higher frequency of being gained in cities relative to natives especially under greater greenhouse gas emissions (S2 File). These findings suggest there are interactions occurring between climate change and species invasion that could act synergistically to threaten urban diversity, although we must caveat these findings that exotic species only represented 1% of our species list. Recent empirical evidence supports Anthropogenically-driven climate change causing shifts in urban species that, in this study, we extend across all terrestrial wildlife, the largest effort of its kind to date.

### Limitations and additional considerations

The taxa negatively affected by climate change in our study are likely to be affected by additional impacts, further reducing their persistence in urban environments. Cities are often stressful for animals, having higher rates of zoonotic diseases [72], habitat fragmentation [73], light and noise pollution [74, 75], pet caused mortality [76], and warmer temperatures [77]. The recent pandemic lockdown in North America produced an increase in bird abundances, suggesting human activity is negatively correlated with urban wildlife [78]. Conversely, some species have evolved adaptations to urban environments [73, 79], potentially overlapping with some degree of resiliency against climate change. Moreover, cities contain many different microclimates and can support a diversity of habitat types through practices such as supplemental irrigation. For instance, urban heat island effects have repeatedly been reported in cities [80, 81] and can have fine-scale variation in air temperatures (<100 m) of as much as 3° C throughout the city [82]. These large temperature differences can function as refugia or introduction points for some species in the larger context of the macroclimatic patterns in the region. However, while some animal species can exist in these islands of climate suitability within select portions of the city, these species will likely be isolated based on the regional climate patterns. Some features of cities may provide temporary refugia for some species, but the additional stressors caused by urbanization coupled with future climate shifts will shrink the available habitat of many species and isolate their remnant populations.

Our results used a climate-only examination for projecting the occurrence of species in cities, but there are many non-climate factors that impact distribution as well. Recent work has found that the predictability of species distribution models can be improved by including species interactions [83], connectivity [84], dispersal [85], and land cover [86]. Our estimates of shifts in urban animal species composition are thus relatively conservative compared to the realized future impact of climate change on the abundance and diversity of wildlife. Predictions of climate suitability are effective at estimating potential declines in occurrence (i.e., species cannot exist outside their climatic niche), but estimated increases in climate suitability may not necessarily translate to an increase in occurrence for the above reasons. These ecological dynamics may result in biodiversity patterns lagging behind expected changes in species composition from climate change [87]. There is accumulating evidence that taxa, such as birds, butterflies, and bees, are experiencing a climate debt and are unable to track a changing climate [17, 68, 87–89], suggesting our results may be downwardly biased in estimates of future biodiversity turnover. Including the effects of non-climate variables in the species distribution modelling could have improved model accuracy, but with over 2000 species are computational prohibited and can be largely speculative. For instance, including species interactions in our models would involve creating a *n*-dimensional matrix for every species with all the trophic and non-trophic interactions for all species we modelled, species we did not model (e.g., plants, fungi), and novel interactions created in the future. We explored the role species interactions may play in impacting the future distribution of urban species using changes in co-occurrence as a proxy and found potentially significant changes in the network of interactions among species (See S3 File for a discussion). Modelling macro-ecological patterns across many taxa and over a large spatial gradient can be informative of general trends expected in the future, but the inclusion of non-climate variables can help improve the accuracy when looking at species and location specific outcomes.

## Conclusion

Our findings identify a *great urban shift* occurring in wildlife across North American cities because of climate change. We believe the relatively short timeframe (i.e., within a few decades) and volume of climate change impacts will produce a dramatic change in many urban species communities. The widespread changes in the representation of wildlife will directly affect the cultural identity, heritage, and symbolism for human residents. The loss of urban biodiversity may also negatively affect mental well-being of residents [90] and the economy (e.g., lost tourism, decreased property aesthetics, more invasive species). The impacts of animal species departing urban areas extend well beyond cultural influences and will likely also include a loss of the ecosystems services they provide [10], such as pest management [91], pollination [71, 92], disease control [91], and decomposition [93]. There is critical need to quantify the consequences of the changes to urban species composition expected to occur in the coming decades, and to develop mitigation strategies to preserve this important biodiversity.

## Supporting information

S1 Fig Analysis workflow of species distribution models. (DOCX)

S2 Fig Projections of species change relative to current climate. (DOCX)

S1 Table Sixty cities examined in Canada and the US. (DOCX)

S2 Table List of all species occurrence datasets. (DOCX)

S3 Table Patterns of contemporary species richness in cities. (DOCX)

S1 File Methods for parameterizing input data into MaxEnt. (DOCX)

S2 File Climate change effects on at-risk and exotic urban species. (DOCX)

S3 File The effect of species interactions in modelling distributions. (DOCX)
