# Supplementary material for: The great urban shift: Climate change is predicted to drive mass species turnover in cities
Source: PLoS One. 2024 Mar 27;19(3):e0299217. doi: 10.1371/journal.pone.0299217 (PMC10971775; doi:10.1371/journal.pone.0299217)
Supplement: S1 Fig — (DOCX) [file pone.0299217.s001.docx]

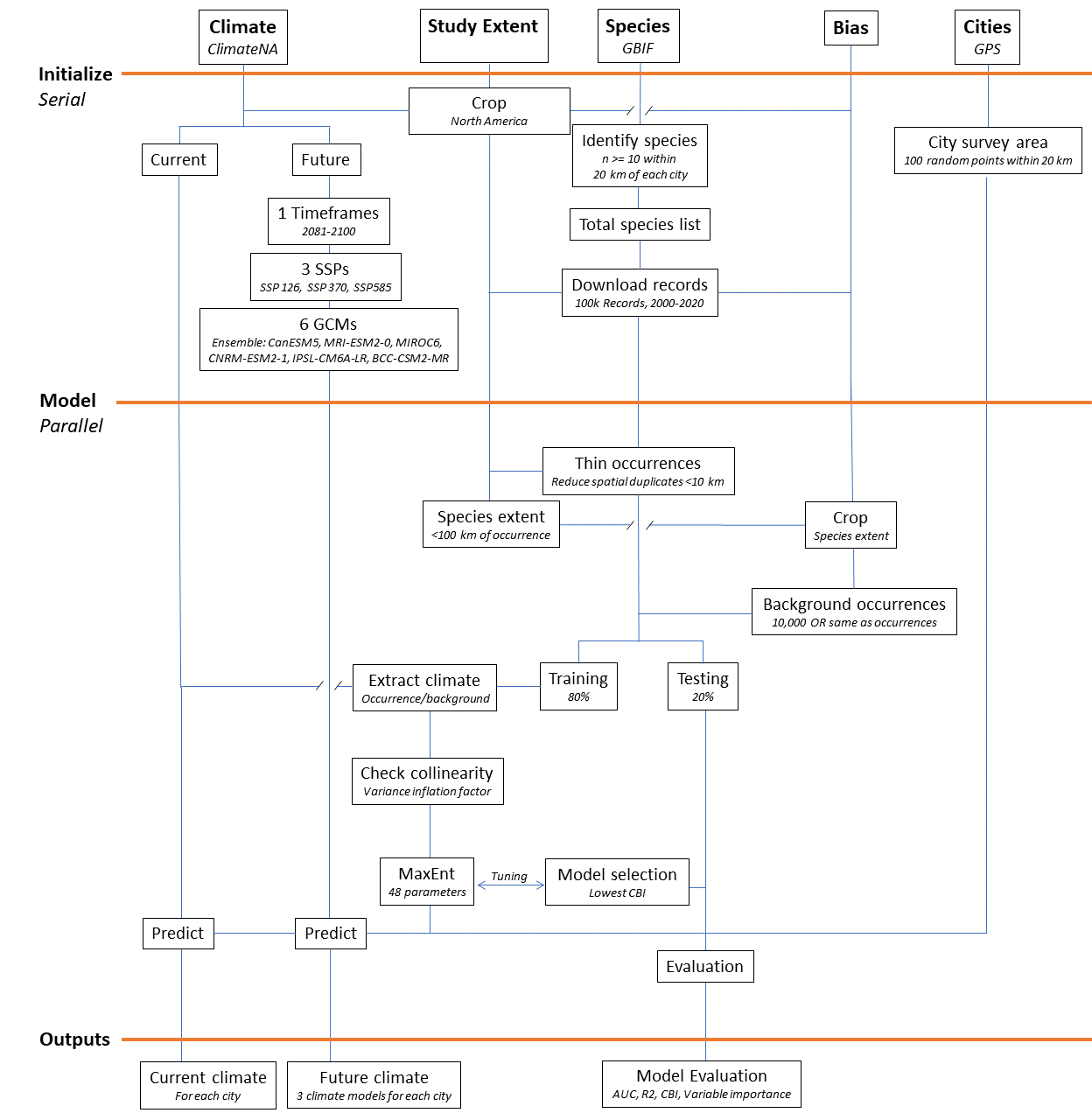


**S1 Fig:** Analysis workflow of species distribution models. Data acquisition, preparation, and analysis workflow for the species distribution modelling of 2,259 species using MaxEnt.
