# Supplementary material for: The great urban shift: Climate change is predicted to drive mass species turnover in cities
Source: PLoS One. 2024 Mar 27;19(3):e0299217. doi: 10.1371/journal.pone.0299217 (PMC10971775; doi:10.1371/journal.pone.0299217)
Supplement: S2 Fig — (DOCX) [file pone.0299217.s002.docx]

| 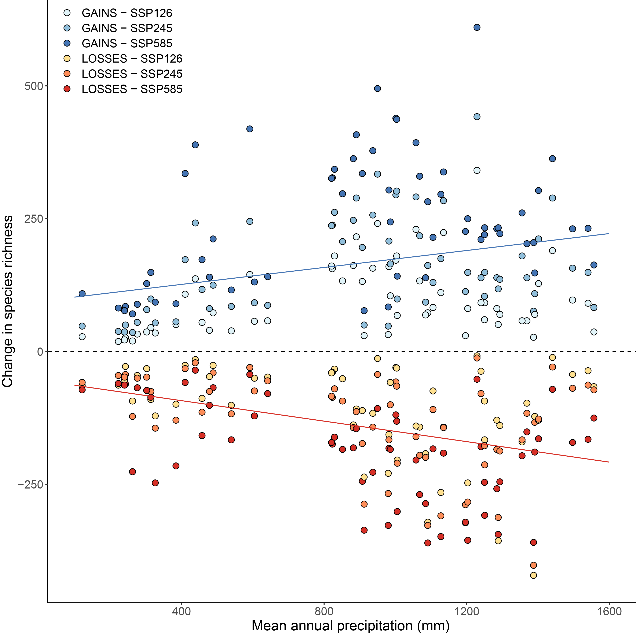 | 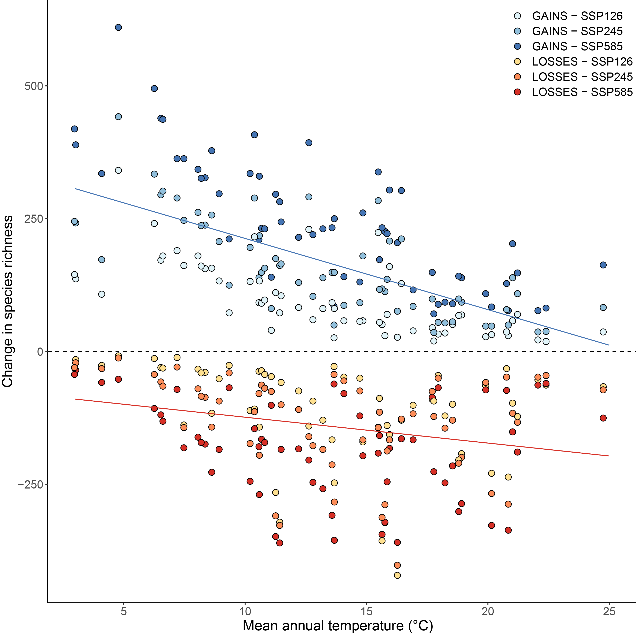 |
| --- | --- |

**S2 Fig:** The relationship between historical climate conditions and the predicted change in species richness for 60 cities in Canada and the USA under three different SSPs.
