# Supplementary material for: The great urban shift: Climate change is predicted to drive mass species turnover in cities
Source: PLoS One. 2024 Mar 27;19(3):e0299217. doi: 10.1371/journal.pone.0299217 (PMC10971775; doi:10.1371/journal.pone.0299217)
Supplement: S1 Table — (DOCX) [file pone.0299217.s003.docx]

**S1 Table:** Sixty cities used for exploring the effects of climate change on urban wildlife. We selected all cities in the Canada or the United States of America that had a population greater than 400,000 people.

| City | Country | Longitude | Latitude | Population (Year) |
| --- | --- | --- | --- | --- |
| *Albuquerque* | USA | -106.65 | 35.09 | 559,121 (2015) |
| *Atlanta* | USA | -84.39 | 33.75 | 506,811 (2019) |
| *Austin* | USA | -97.74 | 30.27 | 931,830 (2015) |
| *Baltimore* | USA | -76.61 | 39.29 | 621,849 (2015) |
| *Boston* | USA | -71.06 | 42.36 | 667,137 (2015) |
| *Brampton* | Canada | -79.77 | 43.68 | 593,638 (2016) |
| *Calgary* | Canada | -114.06 | 51.04 | 1,230,915 (2015) |
| *Charlotte* | USA | -80.83 | 35.21 | 827,097 (2015) |
| *Chicago* | USA | -87.65 | 41.76 | 2,720,546 (2015) |
| *Colorado Springs* | USA | -104.82 | 38.83 | 478,221 (2019) |
| *Columbus* | USA | -82.99 | 39.99 | 850,106 (2015) |
| *Dallas* | USA | -96.8 | 32.78 | 1,300,092 (2015) |
| *Denver* | USA | -104.98 | 39.73 | 682,545 (2015) |
| *Detroit* | USA | -83.07 | 42.33 | 677,116 (2015) |
| *Edmonton* | Canada | -113.47 | 53.56 | 899,447 (2016) |
| *El Paso* | USA | -106.49 | 31.76 | 681,124 (2015) |
| *Fort Worth* | USA | -97.31 | 32.75 | 833,319 (2015) |
| *Fresno* | USA | -119.79 | 36.74 | 520,052 (2015) |
| *Halifax* | USA | -63.6 | 44.68 | 403,131 (2016) |
| *Hamilton* | Canada | -79.82 | 43.26 | 556,359 (2015) |
| *Houston* | USA | -95.37 | 29.76 | 2,296,224 (2015) |
| *Indianapolis* | USA | -86.15 | 39.78 | 853,173 (2015) |
| *Jacksonville* | USA | -81.66 | 30.33 | 868,031 (2015) |
| *Kansas City* | USA | -94.59 | 39.1 | 486,404 (2020) |
| *Las Vegas* | USA | -115.14 | 36.18 | 623,747 (2015) |
| *Laval* | Canada | -73.75 | 45.58 | 422,993 (2016) |
| *Long Beach* | USA | -118.19 | 33.77 | 462,257 (2019) |
| *Los Angeles* | USA | -118.27 | 34.05 | 3,971,883 (2015) |
| *Memphis* | USA | -90.04 | 35.14 | 655,770 (2015) |
| *Mesa* | USA | -111.82 | 33.42 | 518,012 (2019) |
| *Miami* | USA | -80.22 | 25.76 | 467,963 (2019) |
| *Milwaukee* | USA | -87.91 | 43.04 | 600,155 (2015) |
| *Minneapolis* | USA | -93.27 | 44.96 | 429,606 (2019) |
| *Mississauga* | Canada | -79.65 | 43.60 | 721,599 (2016) |
| *Montréal* | Canada | -73.65 | 45.54 | 1,753,034 (2015) |
| *Nashville* | USA | -86.78 | 36.17 | 654,610 (2015) |
| *New York* | USA | -74.01 | 40.71 | 8,550,405 (2015) |
| *Oakland* | USA | -122.3 | 37.81 | 433,031 (2019) |
| *Oklahoma City* | USA | -97.52 | 35.47 | 631,346 (2015) |
| *Omaha* | USA | -95.97 | 41.26 | 478,192 (2019) |
| *Ottawa - Gatineau* | Canada | -75.69 | 45.4 | 956,710 (2015) |
| *Philadelphia* | USA | -75.16 | 39.95 | 1,567,442 (2015) |
| *Phoenix* | USA | -112.07 | 33.45 | 1,563,025 (2015) |
| *Portland* | USA | -122.65 | 45.52 | 632,309 (2015) |
| *Québec* | Canada | -71.29 | 46.82 | 540,994 (2015) |
| *Raleigh* | USA | -78.64 | 35.79 | 474,069 (2019) |
| *Sacramento* | USA | -121.47 | 38.57 | 513,624 (2019) |
| *San Antonio* | USA | -98.49 | 29.42 | 1,469,845 (2015) |
| *San Diego* | USA | -117.15 | 32.72 | 1,394,928 (2015) |
| *San Francisco* | USA | -122.44 | 37.77 | 864,816 (2015) |
| *San Jose* | USA | -121.89 | 37.34 | 1,026,908 (2015) |
| *Seattle* | USA | -122.32 | 47.59 | 684,451 (2015) |
| *Surrey* | Canada | -122.85 | 49.19 | 518,467 (2017) |
| *Toronto* | Canada | -79.48 | 43.73 | 2,826,498 (2015) |
| *Tucson* | USA | -110.97 | 32.22 | 531,641 (2015) |
| *Tulsa* | USA | -95.99 | 36.15 | 401,190 (2019) |
| *Vancouver* | Canada | -122.97 | 49.24 | 648,608 (2015) |
| *Virginia Beach* | USA | -76.06 | 36.75 | 449,974 (2020) |
| *Washington* | USA | -77.03 | 38.89 | 672,228 (2015) |
| *Winnipeg* | Canada | -97.16 | 49.88 | 718,400 (2015) |
