# Supplementary material for: The great urban shift: Climate change is predicted to drive mass species turnover in cities
Source: PLoS One. 2024 Mar 27;19(3):e0299217. doi: 10.1371/journal.pone.0299217 (PMC10971775; doi:10.1371/journal.pone.0299217)
Supplement: S2 Table — (DOCX) [file pone.0299217.s004.docx]

**S2 Table:** A list of all the GBIF downloads to obtain the species occurrences used in the study.

| **Website** | **Date downloaded** | **Type of download** | **DOI** |
| --- | --- | --- | --- |
| GBIF.org | June 4 2021 | GBIF occurrences | https://doi.org/10.15468/dl.bcfewa |
| GBIF.org | June 4 2021 | GBIF occurrences | https://doi.org/10.15468/dl.dqzx92 |
| GBIF.org | June 4 2021 | GBIF occurrences | https://doi.org/10.15468/dl.dqknfc |
| GBIF.org | June 4 2021 | GBIF occurrences | https://doi.org/10.15468/dl.ww83a8 |
| GBIF.org | June 4 2021 | GBIF occurrences | https://doi.org/10.15468/dl.xag8mq |
| GBIF.org | June 4 2021 | GBIF occurrences | https://doi.org/10.15468/dl.rcjxkc |
| GBIF.org | June 4 2021 | GBIF occurrences | https://doi.org/10.15468/dl.ydnc36 |
| GBIF.org | June 4 2021 | GBIF occurrences | https://doi.org/10.15468/dl.asv7tq |
| GBIF.org | June 4 2021 | GBIF occurrences | https://doi.org/10.15468/dl.ks8t9q |
| GBIF.org | June 4 2021 | GBIF occurrences | https://doi.org/10.15468/dl.7bcwwv |
| GBIF.org | June 4 2021 | GBIF occurrences | https://doi.org/10.15468/dl.yr6pkb |
| GBIF.org | June 4 2021 | GBIF occurrences | https://doi.org/10.15468/dl.7sjxxv |
