# Supplementary material for: The great urban shift: Climate change is predicted to drive mass species turnover in cities
Source: PLoS One. 2024 Mar 27;19(3):e0299217. doi: 10.1371/journal.pone.0299217 (PMC10971775; doi:10.1371/journal.pone.0299217)
Supplement: S3 Table — (DOCX) [file pone.0299217.s005.docx]

**S3 Table:** Patterns of contemporary species richness in cities under historic and future climate conditions. We provide the number of species predicted to be gained, lost, or remain unchanged under three future climate scenarios (SSPs) in each of the 60 cities. Species with a predicted occurrence below the threshold identified by the MaxEnt model were considered absent from the respective city.

| **SSP** | **City** | **Gained** | **Lost** | **Unchanged** | **Historic Richness** |
| --- | --- | --- | --- | --- | --- |
| SSP126 | Albuquerque | 33 | 39 | 186 | 225 |
| SSP245 | Albuquerque | 60 | 51 | 174 | 225 |
| SSP585 | Albuquerque | 83 | 64 | 161 | 225 |
| SSP126 | Atlanta | 28 | 451 | 297 | 748 |
| SSP245 | Atlanta | 83 | 427 | 321 | 748 |
| SSP585 | Atlanta | 229 | 381 | 367 | 748 |
| SSP126 | Austin | 28 | 225 | 701 | 926 |
| SSP245 | Austin | 47 | 255 | 671 | 926 |
| SSP585 | Austin | 75 | 324 | 602 | 926 |
| SSP126 | Baltimore | 96 | 176 | 950 | 1126 |
| SSP245 | Baltimore | 157 | 245 | 881 | 1126 |
| SSP585 | Baltimore | 244 | 340 | 786 | 1126 |
| SSP126 | Boston | 105 | 59 | 1048 | 1107 |
| SSP245 | Boston | 152 | 110 | 997 | 1107 |
| SSP585 | Boston | 222 | 220 | 887 | 1107 |
| SSP126 | Calgary | 139 | 38 | 163 | 201 |
| SSP245 | Calgary | 202 | 48 | 153 | 201 |
| SSP585 | Calgary | 354 | 79 | 122 | 201 |
| SSP126 | Charlotte | 42 | 322 | 446 | 768 |
| SSP245 | Charlotte | 119 | 282 | 486 | 768 |
| SSP585 | Charlotte | 251 | 310 | 458 | 768 |
| SSP126 | Chicago | 136 | 169 | 862 | 1031 |
| SSP245 | Chicago | 218 | 209 | 822 | 1031 |
| SSP585 | Chicago | 327 | 319 | 712 | 1031 |
| SSP126 | Colorado Springs | 65 | 41 | 375 | 416 |
| SSP245 | Colorado Springs | 107 | 75 | 341 | 416 |
| SSP585 | Colorado Springs | 186 | 127 | 289 | 416 |
| SSP126 | Columbus | 88 | 322 | 462 | 784 |
| SSP245 | Columbus | 187 | 325 | 459 | 784 |
| SSP585 | Columbus | 311 | 373 | 411 | 784 |
| SSP126 | Dallas | 76 | 196 | 723 | 919 |
| SSP245 | Dallas | 101 | 218 | 701 | 919 |
| SSP585 | Dallas | 143 | 289 | 630 | 919 |
| SSP126 | Denver | 51 | 63 | 240 | 303 |
| SSP245 | Denver | 96 | 85 | 218 | 303 |
| SSP585 | Denver | 148 | 120 | 183 | 303 |
| SSP126 | Detroit | 148 | 141 | 873 | 1014 |
| SSP245 | Detroit | 222 | 191 | 823 | 1014 |
| SSP585 | Detroit | 341 | 293 | 721 | 1014 |
| SSP126 | Edmonton | 152 | 22 | 117 | 139 |
| SSP245 | Edmonton | 264 | 32 | 107 | 139 |
| SSP585 | Edmonton | 412 | 54 | 85 | 139 |
| SSP126 | El Paso | 38 | 56 | 186 | 242 |
| SSP245 | El Paso | 59 | 69 | 173 | 242 |
| SSP585 | El Paso | 98 | 87 | 155 | 242 |
| SSP126 | Fort Worth | 79 | 210 | 704 | 914 |
| SSP245 | Fort Worth | 106 | 226 | 688 | 914 |
| SSP585 | Fort Worth | 144 | 297 | 617 | 914 |
| SSP126 | Fresno | 48 | 80 | 435 | 515 |
| SSP245 | Fresno | 95 | 71 | 444 | 515 |
| SSP585 | Fresno | 149 | 89 | 426 | 515 |
| SSP126 | Halifax | 216 | 19 | 687 | 706 |
| SSP245 | Halifax | 317 | 30 | 676 | 706 |
| SSP585 | Halifax | 410 | 70 | 636 | 706 |
| SSP126 | Hamilton | 147 | 56 | 947 | 1003 |
| SSP245 | Hamilton | 237 | 97 | 906 | 1003 |
| SSP585 | Hamilton | 320 | 206 | 797 | 1003 |
| SSP126 | Houston | 79 | 125 | 952 | 1077 |
| SSP245 | Houston | 121 | 148 | 929 | 1077 |
| SSP585 | Houston | 157 | 204 | 873 | 1077 |
| SSP126 | Indianapolis | 134 | 274 | 522 | 796 |
| SSP245 | Indianapolis | 206 | 311 | 485 | 796 |
| SSP585 | Indianapolis | 323 | 362 | 434 | 796 |
| SSP126 | Jacksonville | 61 | 96 | 918 | 1014 |
| SSP245 | Jacksonville | 117 | 131 | 883 | 1014 |
| SSP585 | Jacksonville | 191 | 193 | 821 | 1014 |
| SSP126 | Kansas City | 229 | 148 | 504 | 652 |
| SSP245 | Kansas City | 291 | 169 | 483 | 652 |
| SSP585 | Kansas City | 382 | 210 | 442 | 652 |
| SSP126 | Las Vegas | 24 | 63 | 240 | 303 |
| SSP245 | Las Vegas | 49 | 63 | 240 | 303 |
| SSP585 | Las Vegas | 121 | 80 | 223 | 303 |
| SSP126 | Long Beach | 42 | 169 | 1094 | 1263 |
| SSP245 | Long Beach | 52 | 194 | 1069 | 1263 |
| SSP585 | Long Beach | 89 | 306 | 957 | 1263 |
| SSP126 | Los Angeles | 42 | 158 | 1093 | 1251 |
| SSP245 | Los Angeles | 55 | 192 | 1059 | 1251 |
| SSP585 | Los Angeles | 79 | 298 | 953 | 1251 |
| SSP126 | Memphis | 125 | 122 | 385 | 507 |
| SSP245 | Memphis | 219 | 106 | 401 | 507 |
| SSP585 | Memphis | 287 | 145 | 362 | 507 |
| SSP126 | Mesa | 19 | 77 | 311 | 388 |
| SSP245 | Mesa | 40 | 75 | 313 | 388 |
| SSP585 | Mesa | 95 | 81 | 307 | 388 |
| SSP126 | Miami | 33 | 90 | 1106 | 1196 |
| SSP245 | Miami | 88 | 88 | 1108 | 1196 |
| SSP585 | Miami | 148 | 150 | 1046 | 1196 |
| SSP126 | Milwaukee | 194 | 119 | 729 | 848 |
| SSP245 | Milwaukee | 292 | 157 | 691 | 848 |
| SSP585 | Milwaukee | 411 | 259 | 589 | 848 |
| SSP126 | Minneapolis | 175 | 158 | 394 | 552 |
| SSP245 | Minneapolis | 265 | 151 | 401 | 552 |
| SSP585 | Minneapolis | 379 | 196 | 356 | 552 |
| SSP126 | Montréal | 188 | 35 | 649 | 684 |
| SSP245 | Montréal | 314 | 69 | 615 | 684 |
| SSP585 | Montréal | 496 | 144 | 540 | 684 |
| SSP126 | Nashville | 76 | 171 | 281 | 452 |
| SSP245 | Nashville | 215 | 157 | 295 | 452 |
| SSP585 | Nashville | 296 | 189 | 263 | 452 |
| SSP126 | New York | 61 | 114 | 1053 | 1167 |
| SSP245 | New York | 109 | 189 | 978 | 1167 |
| SSP585 | New York | 240 | 274 | 893 | 1167 |
| SSP126 | Oakland | 72 | 70 | 1324 | 1394 |
| SSP245 | Oakland | 109 | 107 | 1287 | 1394 |
| SSP585 | Oakland | 145 | 182 | 1212 | 1394 |
| SSP126 | Oklahoma City | 153 | 145 | 448 | 593 |
| SSP245 | Oklahoma City | 198 | 155 | 438 | 593 |
| SSP585 | Oklahoma City | 284 | 169 | 424 | 593 |
| SSP126 | Omaha | 225 | 112 | 301 | 413 |
| SSP245 | Omaha | 297 | 122 | 291 | 413 |
| SSP585 | Omaha | 397 | 152 | 261 | 413 |
| SSP126 | Ottawa - Gatineau | 235 | 26 | 597 | 623 |
| SSP245 | Ottawa - Gatineau | 347 | 58 | 565 | 623 |
| SSP585 | Ottawa - Gatineau | 521 | 134 | 489 | 623 |
| SSP126 | Philadelphia | 91 | 148 | 932 | 1080 |
| SSP245 | Philadelphia | 150 | 202 | 878 | 1080 |
| SSP585 | Philadelphia | 256 | 300 | 780 | 1080 |
| SSP126 | Phoenix | 28 | 78 | 284 | 362 |
| SSP245 | Phoenix | 53 | 62 | 300 | 362 |
| SSP585 | Phoenix | 110 | 75 | 287 | 362 |
| SSP126 | Portland | 86 | 83 | 1183 | 1266 |
| SSP245 | Portland | 153 | 131 | 1135 | 1266 |
| SSP585 | Portland | 231 | 221 | 1045 | 1266 |
| SSP126 | Québec | 361 | 8 | 458 | 466 |
| SSP245 | Québec | 485 | 15 | 451 | 466 |
| SSP585 | Québec | 649 | 58 | 408 | 466 |
| SSP126 | Raleigh | 46 | 363 | 496 | 859 |
| SSP245 | Raleigh | 124 | 313 | 546 | 859 |
| SSP585 | Raleigh | 240 | 346 | 513 | 859 |
| SSP126 | Sacramento | 66 | 123 | 659 | 782 |
| SSP245 | Sacramento | 105 | 135 | 647 | 782 |
| SSP585 | Sacramento | 122 | 170 | 612 | 782 |
| SSP126 | San Antonio | 38 | 232 | 632 | 864 |
| SSP245 | San Antonio | 58 | 270 | 594 | 864 |
| SSP585 | San Antonio | 81 | 323 | 541 | 864 |
| SSP126 | San Diego | 31 | 128 | 1219 | 1347 |
| SSP245 | San Diego | 47 | 178 | 1169 | 1347 |
| SSP585 | San Diego | 73 | 303 | 1044 | 1347 |
| SSP126 | San Francisco | 75 | 42 | 1353 | 1395 |
| SSP245 | San Francisco | 92 | 51 | 1344 | 1395 |
| SSP585 | San Francisco | 164 | 94 | 1301 | 1395 |
| SSP126 | San Jose | 59 | 136 | 975 | 1111 |
| SSP245 | San Jose | 122 | 161 | 950 | 1111 |
| SSP585 | San Jose | 166 | 220 | 891 | 1111 |
| SSP126 | Seattle | 110 | 79 | 1241 | 1320 |
| SSP245 | Seattle | 178 | 142 | 1178 | 1320 |
| SSP585 | Seattle | 266 | 233 | 1087 | 1320 |
| SSP126 | Toronto | 159 | 40 | 848 | 888 |
| SSP245 | Toronto | 246 | 94 | 794 | 888 |
| SSP585 | Toronto | 360 | 195 | 693 | 888 |
| SSP126 | Tucson | 37 | 42 | 320 | 362 |
| SSP245 | Tucson | 79 | 55 | 307 | 362 |
| SSP585 | Tucson | 132 | 84 | 278 | 362 |
| SSP126 | Tulsa | 221 | 137 | 474 | 611 |
| SSP245 | Tulsa | 285 | 144 | 467 | 611 |
| SSP585 | Tulsa | 325 | 187 | 424 | 611 |
| SSP126 | Vancouver | 119 | 38 | 1307 | 1345 |
| SSP245 | Vancouver | 192 | 82 | 1263 | 1345 |
| SSP585 | Vancouver | 272 | 185 | 1160 | 1345 |
| SSP126 | Virginia Beach | 78 | 147 | 1002 | 1149 |
| SSP245 | Virginia Beach | 168 | 203 | 946 | 1149 |
| SSP585 | Virginia Beach | 257 | 286 | 863 | 1149 |
| SSP126 | Washington | 83 | 270 | 760 | 1030 |
| SSP245 | Washington | 174 | 292 | 738 | 1030 |
| SSP585 | Washington | 265 | 375 | 655 | 1030 |
| SSP126 | Winnipeg | 164 | 31 | 118 | 149 |
| SSP245 | Winnipeg | 273 | 34 | 115 | 149 |
| SSP585 | Winnipeg | 452 | 50 | 99 | 149 |
