# Supplementary material for: The great urban shift: Climate change is predicted to drive mass species turnover in cities
Source: PLoS One. 2024 Mar 27;19(3):e0299217. doi: 10.1371/journal.pone.0299217 (PMC10971775; doi:10.1371/journal.pone.0299217)
Supplement: S1 File — (DOCX) [file pone.0299217.s006.docx]

**S1 Supplemental –** **Methods for parameterizing input data into MaxEnt**

For each species with fewer than 10,000 occurrence records in our study area (i.e., North America), we selected 10,000 background points to capture the range of climate conditions to compare to the occurrence data. Background points were randomly selected from an area within 100 km of the occurrence records (function *randomPoints*, package *dismo* (Hijmans *et al.* 2017). For all species greater than 10,000 occurrence records, we used the same number of occurrence records as the background points (i.e., 1:1 ratio). Although some studies use a 10:1 ratio of background points to occurrence records (e.g. Filazzola *et al.* 2020), there has been evidence that this ratio does not lead to model improvement with records greater than 10,000 observations (Phillips & Dudík 2008). We did not restrict the geographic distribution of any occurrence records beyond these steps, thus different observations may represent the same species reported at different seasonal localities (e.g., summer vs. winter range for migratory birds or butterflies).

To minimize the impact of spatial autocorrelation, we thinned occurrences at a resolution greater than the grid cells of the climate data (i.e., 5 km vs 800 m), removed collinear climate variables, and selected model tuning that considered a spatial component of the environmental variables. Coarse thinning of observations reduces clustering of occurrences leading to a more uniform distribution, reducing inherent autocorrelation. To remove collinear variables, we extracted the historic climate conditions for the occurrence and background points for each species and tested for collinearity among our pre-selected climate variables (function *vifcor*, package *usdm*) (Naimi *et al.* 2014). Variables identified to have a high variance inflation factor (e.g., VI > 10) were excluded from species distribution modelling (Naimi *et al.* 2014). Finally, for model tuning we used spatial block cross-validation (partitions = “block” in package *ENMeval*) (Muscarella *et al.* 2014). Rather than using random subsets of occurrence and background records for tuning (e.g., random k-folds), spatial block cross-validation creates spatially separated training and testing datasets to evaluate model accuracy at different scales and spatial groupings, thereby minimizing overfitting of training data (Dormann, Carsten *et al.* 2007; Valavi *et al.* 2019). Thinning, identification of co-linear variables, and model tuning using spatial block cross-validation was applied to each species independently.

**References**

Dormann, Carsten, F., McPherson, Jana, M., Araújo, Miguel, B., Bivand, R., Bolliger, J., Carl, G., *et al.* (2007). Methods to account for spatial autocorrelation in the analysis of species distributional data: a review. *Ecography (Cop.).*, 30, 609–628.

Filazzola, A., Matter, S.F. & Roland, J. (2020). Inclusion of trophic interactions increases the vulnerability of an alpine butterfly species to climate change. *Glob. Chang. Biol.*, 26, 2867–2877.

Hijmans, R.J., Phillips, S., Leathwick, J., Elith, J. & Hijmans, M.R.J. (2017). Package ‘dismo.’ *Circles*, 9, 1–68.

Lenth, R., Singmann, H., Love, J., Buerkner, P. & Herve, M. (2018). Emmeans: Estimated marginal means, aka least-squares means. *R Packag. version*, 1, 3.

Muscarella, R., Galante, P.J., Soley-Guardia, M., Boria, R.A., Kass, J., Uriarte, M., *et al.* (2014). ENMeval: An R package for conducting spatially independent evaluations and estimating optimal model complexity for ecological niche models. *Methods Ecol. Evol.*, 5, 1198–1205.

Naimi, B., Hamm, N.A.S., Groen, T.A., Skidmore, A.K. & Toxopeus, A.G. (2014). Where is positional uncertainty a problem for species distribution modelling? *Ecography (Cop.).*, 37, 191–203.

Phillips, S.J. & Dudík, M. (2008). Modeling of species distributions with Maxent: new extensions and a comprehensive evaluation. *Ecography (Cop.).*, 31, 161–175.

Valavi, R., Elith, J., Lahoz-Monfort, J.J. & Guillera-Arroita, G. (2019). blockCV: An r package for generating spatially or environmentally separated folds for k-fold cross-validation of species distribution models. *Methods Ecol. Evol.*, 10, 225–232.
