# Supplementary material for: The great urban shift: Climate change is predicted to drive mass species turnover in cities
Source: PLoS One. 2024 Mar 27;19(3):e0299217. doi: 10.1371/journal.pone.0299217 (PMC10971775; doi:10.1371/journal.pone.0299217)
Supplement: S2 File — (DOCX) [file pone.0299217.s007.docx]

**S2 Supplemental –** **Climate change effects on at-risk urban species**

To determine if species already at-risk of population declines were more likely to be impacted, we compared our list of target species to reports by the International Union for Conservation of Nature (IUCN). We obtained all available IUCN Red List assessments for our species (908 in total). We simplified the IUCN classification into least concern and at-risk (critically endangered, endangered, vulnerable, and near threatened) while omitting three species that were data deficient (*Bombus cryptarum, Heterotilapia buttikoferi, Zapteryx exasperata*). To test if there was a significant difference in the species gained or lost within the two categories of IUCNs status, we fit GLMs separately for at-risk species and least concern species. Each GLM had the number of species as the response and the change (gain, loss, no change) crossed with the SSP scenario as predictors. To determine the SSP scenario with the greatest change, we computed estimated marginal means (package *emmeans*, function *emmeans*)(Lenth *et al.* 2018) to examine all pairwise comparisons between the three SSPs and three change types. We also summarized patterns of gains and loses between natives and exotic species. In total we had 93 species identified as exotic to North America based out of all the species we analyzed.

The patterns of change were consistent between at-risk and Least Concern species (Fig. A), for species that have been assessed by the IUCN Red List of Threatened Species ([www.iucnredlist.org/](http://www.iucnredlist.org/)). We found that at-risk species (i.e., not classified as Least Concern) were predicted to have significantly more turnover among cities in SSP scenarios with more development and greenhouse gas emissions (χ^2^ _4,495_ = 37.4, p < 0.0001; Fig. 4). Similarly, Least Concern species were also found to have greater turnover across cities under the more extreme SSP scenarios (χ^2^ _4,495_ = 55.3, p < 0.0001; Fig. 4). We saw that exotic species were more likely to be gained in cities, and less likely to be lost proportionally to native species (Figure B, Table S2.A).


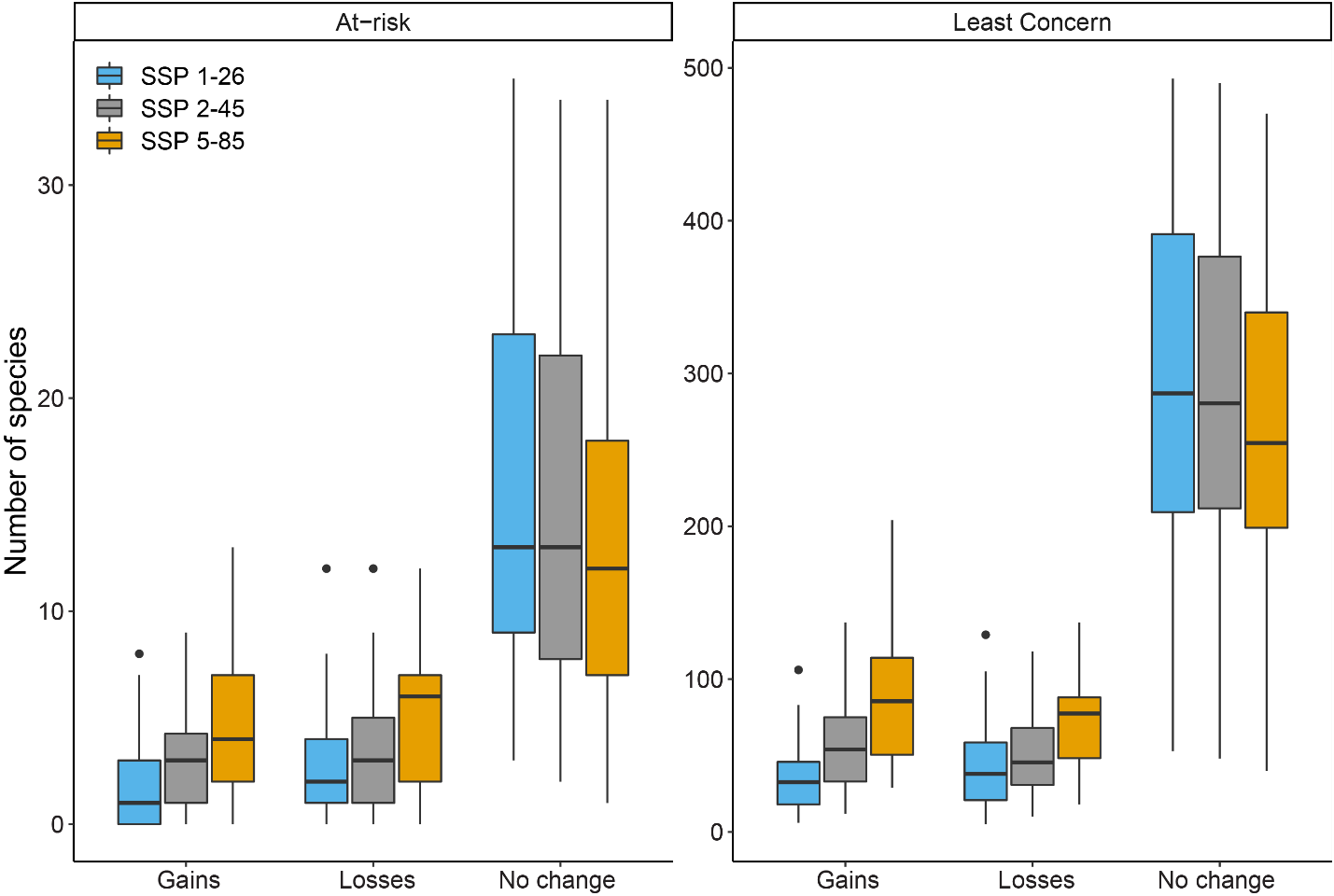


**Figure A:** The average change in species richness among the 60 cities separated by type of change (gains, losses, or no change), IUCN status (at-risk, or least-concern), and shared socio-economic pathway (SSP). In SSP scenarios with greater development and greenhouse gas emissions the number of species affected increased, either resulting in a loss or gain in species richness for the tested cities. We used 905 species for this comparison because not all tested species within our dataset have been assessed by the IUCN.


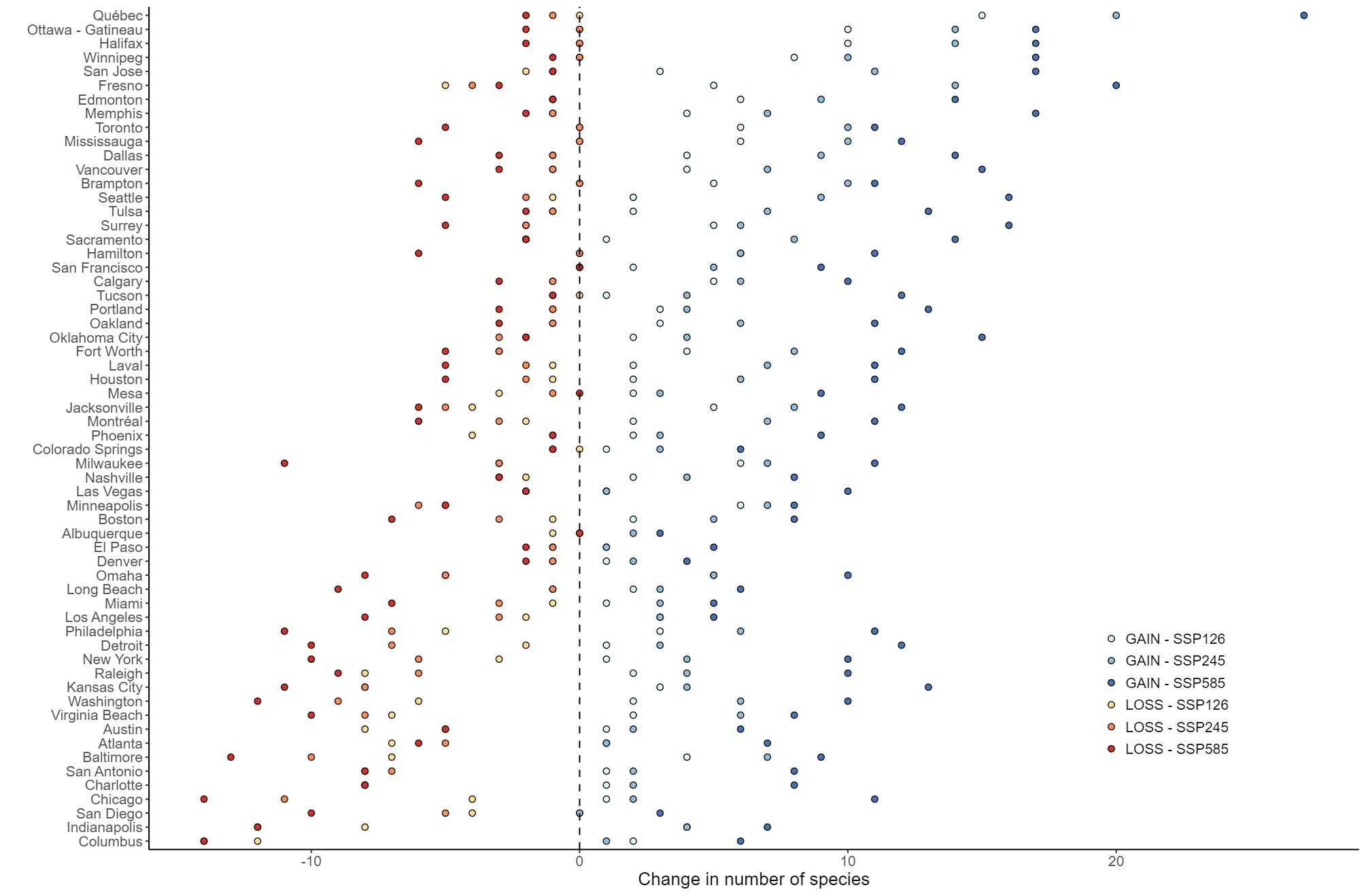


**Figure B:** The total number of gains and losses for exotic species only in each city separated by SSP scenario. Cities at the top of the figure are predicted to have the greatest increase in species richness relative to species’ historical distribution.

**Table S2.A:** The average gains and losses for cities in Canada and the USA separated by native status and SSP scenario.

| **Status** | **SSP** | **Average Gains** | **Average Losses** |
| --- | --- | --- | --- |
| Exotic | ssp126 | 3.28 | 2.83 |
| Exotic | ssp245 | 5.87 | 3.35 |
| Exotic | ssp585 | 10.87 | 5.42 |
| Native | ssp126 | 93.8 | 105.08 |
| Native | ssp245 | 150.3 | 124.12 |
| Native | ssp585 | 229.83 | 173.27 |
