# Supplementary material for: The great urban shift: Climate change is predicted to drive mass species turnover in cities
Source: PLoS One. 2024 Mar 27;19(3):e0299217. doi: 10.1371/journal.pone.0299217 (PMC10971775; doi:10.1371/journal.pone.0299217)
Supplement: S3 File — (DOCX) [file pone.0299217.s008.docx]

**S3 Supplemental – A discussion of the effect of species interactions in modelling distributions**

With the loss and gains of species throughout cities in Canada and the US from climate change there will also be significant changes in the number of species interactions. Determining all the trophic or non-trophic and direct or indirect interactions occurring between species is challenging when the biology of the target species is unknown. For the 2,019 species in our analysis quantifying all interactions is impractical. However, we can use the co-occurrence of species in a city as a proxy for potential interactions and familiarity between species. Based on our predicted occurrence of species for the 60 targeted cities, we generated co-occurrence matrices for historical and future climate conditions. We then calculated the difference between these co-occurrence matrices to determine whether an interaction was lost (two species no longer coexist in the same city) or gained (two species coexist in a city they had not previously).

Many cities are predicted to experience hundreds of new and hundreds of lost coexisting species (Figure S4). These patterns closely follow predictions of overall species change (Table S3). For example, Quebec City is expected to have on average 600-800 new co-occurrences between species depending on the SSP scenario (Figure S4)., largely driven by 361-649 new species that are predicted to occur in the future (Table S3). Similarly, a city such as Atlanta will have a significant loss in species co-occurrences (Figure S4) paralleling the expected decline in resident species (Table S3). Although changes in co-occurrence do not equate to changes in species interactions, these findings demonstrate the potential cascading effects in the ecological communities of these cities that is expected to occur as new species move in and resident species are extirpated.


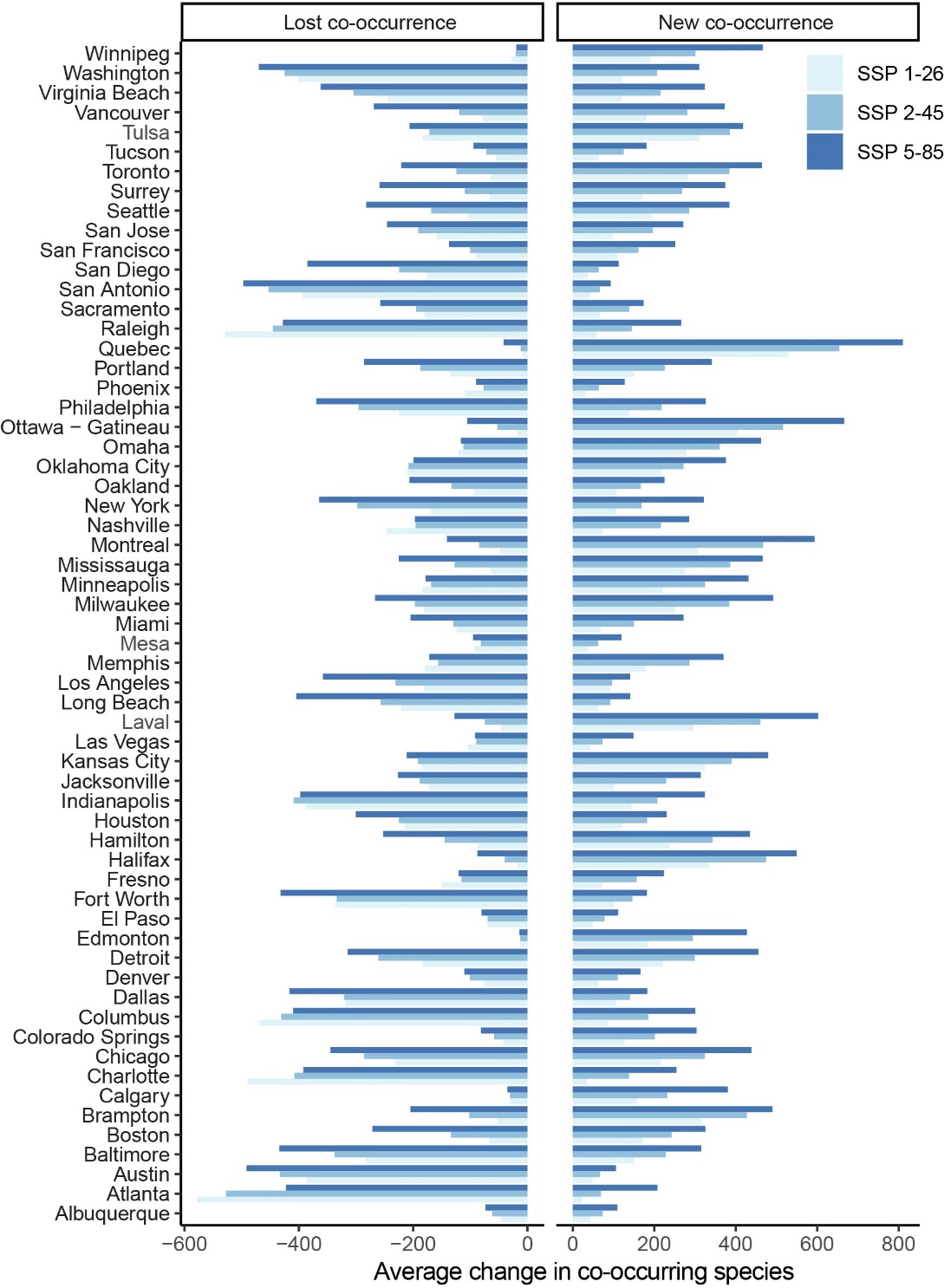


**Figure S5:** The average change in co-occurring species per species in the 60 target cities. Lost co-occurrence represents a species no longer co-occurring with another species for a specific city. A new co-occurrence represents a new co-occurrence between two species.
